# Updates in the chronic kidney disease-mineral bone disorder show the role of osteocytic proteins, a potential mechanism of the bone—Vascular paradox, a therapeutic target, and a biomarker

**DOI:** 10.3389/fphys.2023.1120308

**Published:** 2023-01-26

**Authors:** Matthew J. Williams, Sarah C. White, Zachary Joseph, Keith A. Hruska

**Affiliations:** ^1^ Division of Pediatric Nephrology, Department of Pediatrics, Washington University, Saint Louis, MO, United States; ^2^ Departments of Medicine and Cell Biology, Washington University, Saint Louis, MO, United States

**Keywords:** chronic kidney disease, chronic kidney disease-mineral bone disorder, vascular calcification, renal osteodystrophy, sclerostin (SOST), klotho, fibroblast growth factor 23, parathyroid hormone

## Abstract

The chronic kidney disease-mineral bone disorder (CKD-MBD) is a complex multi-component syndrome occurring during kidney disease and its progression. Here, we update progress in the components of the syndrome, and synthesize recent investigations, which suggest a potential mechanism of the bone-vascular paradox. The discovery that calcified arteries in chronic kidney disease inhibit bone remodeling lead to the identification of factors produced by the vasculature that inhibit the skeleton, thus providing a potential explanation for the bone-vascular paradox. Among the factors produced by calcifying arteries, sclerostin secretion is especially enlightening. Sclerostin is a potent inhibitor of bone remodeling and an osteocyte specific protein. Its production by the vasculature in chronic kidney disease identifies the key role of vascular cell osteoblastic/osteocytic transdifferentiation in vascular calcification and renal osteodystrophy. Subsequent studies showing that inhibition of sclerostin activity by a monoclonal antibody improved bone remodeling as expected, but stimulated vascular calcification, demonstrate that vascular sclerostin functions to brake the Wnt stimulation of the calcification milieu. Thus, the target of therapy in the chronic kidney disease-mineral bone disorder is not inhibition of sclerostin function, which would intensify vascular calcification. Rather, decreasing sclerostin production by decreasing the vascular osteoblastic/osteocytic transdifferentiation is the goal. This might decrease vascular calcification, decrease vascular stiffness, decrease cardiac hypertrophy, decrease sclerostin production, reduce serum sclerostin and improve skeletal remodeling. Thus, the therapeutic target of the chronic kidney disease-mineral bone disorder may be vascular osteoblastic transdifferentiation, and sclerostin levels may be a useful biomarker for the diagnosis of the chronic kidney disease-mineral bone disorder and the progress of its therapy.

## Introduction

**FIGURE 1:**
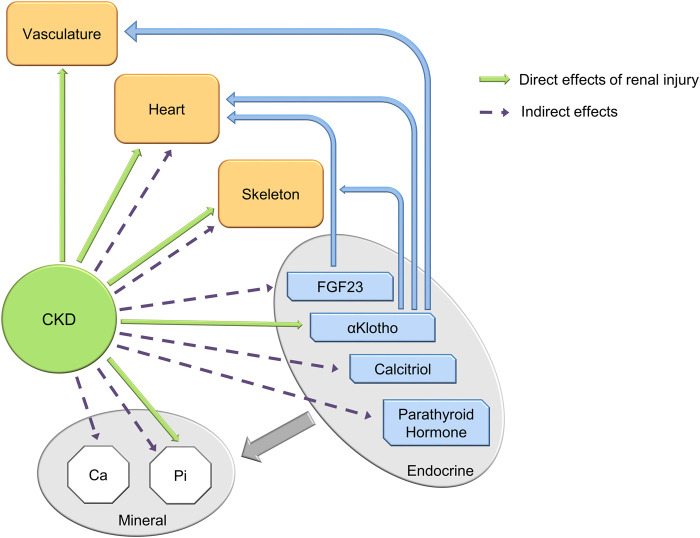
The components of the CKD-MBD— 3 organ dysfunctions, 4 endocrine factors and 2 mineral metabolism dysfunctions. Green arrows show direct effects of renal injury/repair on individual syndrome components, dotted arrows indirect effects. Thick gray arrow indicates that each of the endocrine components regulate mineral homeostasis. Blue arrows show effects of FGF23 and αklotho on organ dysfunction.

**FIGURE 2:**
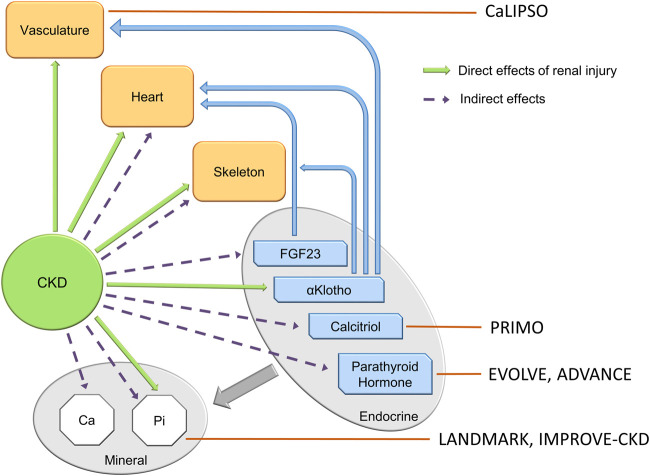
The components of the CKD-MBD, and the targets of clinical trials of attempted therapy. Clinical trials in the CKD-MBD to date have targeted late components of the syndrome, and have not altered cardiovascular outcomes. Small observational trials of romosozumab targeting the skeleton in dialysis patients (not shown in the figure) reveal increased bone mineral density at lumbar and femoral bone sites at 12 and 24 mos. See section on bone—vascular paradox.

The Chronic Kidney Disease–Mineral Bone Disorder (CKD-MBD) is an important contributor to the mortality associated with CKD. Matsushita et al. (2015); Block et al. (2013) Specifically, the CKD-MBD contributes to cardiovascular mortality, the major cause of death in patients with CKD; Go et al. (2004); Tonelli et al. (2006); Navaneethan et al. (2015) The CKD-MBD is a multi-component syndrome consisting of: disordered mineral metabolism, hyperphosphatemia, calcitriol and vitamin D deficiency, secondary hyperparathyroidism, elevated fibroblast growth factor 23 (FGF23), Klotho deficiency, renal osteodystrophy, vascular calcification and cardiac disease, complicating CKD (Figure 1). Hruska et al. (2022) Up to the present - 2022, treatment of the CKD-MBD has focused on the components that develop late in the course of the syndrome. That is, nephrologists use phosphate binders, calcimimetics and calcitriol to control hyperphosphatemia, secondary hyperparathyroidism and treat renal osteodystrophy. Inhibitors of hydroxyapatite are proposed to approach vascular calcification. Raggi et al. (2020) However, these treatments have not affected cardiovascular outcomes in clinical trials (Figure 2).Raggi et al. (2011), Investigators et al. (2012), Raggi et al. (2020), Ogata et al. (2021), Sutherland et al. (2022) This raises the question as to the appropriate outcome of trials to treat the CKD-MBD. Hedayati (2020) As a result, the CKD-MBD is a syndrome without an effective therapy, and in need of therapeutic target identification. Additionally, the field needs biomarker development to guide diagnosis, treatment and prognosis.

**FIGURE 3:**
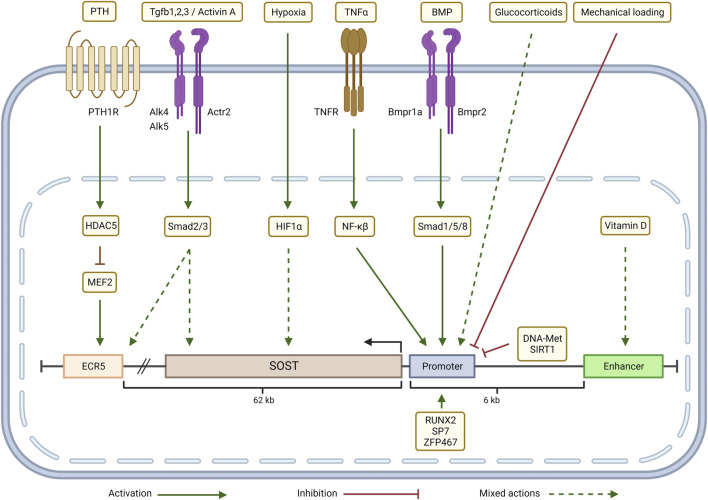
Transcriptional regulation of SOST in the CKD-MBD context: Parathyroid hormone (PTH) inhibits SOST expression through a HDAC5 effect on MEF2 which regulates ECR5. Depending on the cellular context, TGFβ/Activin signaling (via Smad2/3) either up-regulates SOST expression by acting through ECR5 or down-regulates SOST. Hypoxia (via HIF-1α), glucocorticoids and Vitamin D up- or down-regulate SOST expression in a context-dependent manner. TNFα, through a NF-κβ dependent mechanism, and BMP signaling, via Smad1/5/8, increase SOST transcription by targeting the promoter. SOST transcription is negatively regulated by mechanical loading, promoter methylation (DNA-Met) and by epigenetic modifications induced by SIRT1. Zfp467 and transcription factors RUNX2 and SP7 bind to the SOST promoter and activate SOST expression. Adapted from Sebastian and Loots (Bone 96: 76-84, 2018). Graphic created with BioRender.com

The finding that in the face of normal Ca, Pi, calcitriol, and PTH, CKD still produced renal osteodystrophy lead to the hypothesis that renal injury/repair factors released to the circulation produce systemic complications of CKD. Lund et al. (2004) Renal development factors reactivated during injury and attempted repair in kidney disease include members of the Wnt and TGFβ families (Surendran et al., 2002; Surendran et al., 2005; Lima et al., 2019; Malik et al., 2020). Reactivation of Wnts and Activin (TGFβ family) transcriptionally stimulate the family of Wnt inhibitors in the process of attempted repair and progression of disease (Niida et al., 2004; Kamiya et al., 2010; Loots et al., 2012; Edeling et al., 2016; Sebastian and Loots, 2017; Zuo and Liu, 2018; Malik et al., 2020). For example, TGFβ and Activin stimulate sclerostin expression (Figure 3). Additionally, other Wnt and BMP inhibitors such as Sostdc1 (also known as USAG-1 and Wise) are induced in diseased kidneys. Yanagita et al. (2004); Yanagita et al. (2006) The Wnt and BMP inhibitors appear in the circulation and contribute to the pathogenesis of renal osteodystrophy (Surendran et al., 2005; Sabbagh et al., 2012; Fang et al., 2014a; Graciolli et al., 2017). The Wnt inhibitor family members produced in kidney disease include sclerostin, Sostdc1, DKK1 (Dickkopf 1), and SFRP4 (Soluble frizzled related protein 4). Surendran et al. (2005); Hruska et al. (2017) In relation to CKD, Dkk1 and sclerostin have been studied, and even though both inhibit the Wnt ligand–LRP5/6–Frizzled interaction, their downstream responses are different (Evenepoel et al., 2015; Behets et al., 2017). In CKD patients, the association of serum Dkk1 with mineral and bone parameters is minimal, while sclerostin strongly associates (Cejka et al., 2011; Behets et al., 2017). There is a consensus that, activation of Wnts and TGFβ family members (Activin A) during kidney diseases produce circulating Wnt inhibitors, inhibiting skeletal remodeling and contributing early in disease to the development of the CKD-MBD.

Here, in providing updates on the CKD-MBD components, focusing on vascular calcification and the concept of kidney disease produced disruption of homeostasis; the bone vascular paradox is potentially explained. The synthesis of recent studies involving Wnt inhibitors produces the suggestion of the putative CKD-MBD therapeutic target and the identification of sclerostin levels as a useful biomarker for diagnosis and progress of therapy.

## Updates in the chronic kidney disease-mineral bone disorder components

### Vascular

Vascular calcification (VC) has been extensively studied since its addition to the CKD-MBD. Moe et al. (2006), Olgaard and Olgaard (2006) Numerous investigators show that it is an organized cellular based program rather than a passive process; that reduction of inhibitors is cellular function based; and that VC is related to autophagy and matrix vesicles. There are many excellent reviews, including recent ones (Paloian and Giachelli, 2014; Lee et al., 2020; Nelson et al., 2020; Lanzer et al., 2021). The science shows that vascular calcification is an important factor in the progression of CKD and the development of cardiac complications of CKD (Yun et al., 2022). The pathogenesis of vascular calcification in CKD is complex, and pathologically, is of two types, neointimal and arterial medial (Shanahan et al., 2011; Lanzer et al., 2021). The pathogenesis of atherosclerotic neointimal and arterial medial calcification involves activation of an osteoblastic differentiation program in cells of the neointima of atherosclerotic plaques and in cells of arterial media (Shanahan et al., 1994; Demer, 1995). In translational models of atherosclerosis and diabetes, mild renal insufficiency (equivalent to human stage 2 CKD) reduces the levels of aortic proteins involved in the contractile apparatus of smooth muscle. Fang et al. (2014b) This is in agreement with evidence that vascular smooth muscle cells move into a dedifferentiated synthetic state from their normal contractile differentiated state in disease (Towler et al., 1998; Kokubo et al., 2009). Thus, early in kidney disease arterial smooth muscle dedifferentiation is present (Fang et al., 2014b; Monroy et al., 2015). Dedifferentiated vascular smooth muscle cells are susceptible to lineage transition within the developmental programs of mesenchymal stem cells and early progenitor cells, including the lineage program of osteoblasts. Osteoblastic transition of cells in the arterial walls produces CKD stimulated calcification of atherosclerotic plaques and the vascular media (Giachelli et al., 1993; Shanahan et al., 1994; Shanahan et al., 1999; Moe et al., 2002; Davies et al., 2003; Williams et al., 2018). In addition, vascular cell osteoblastic differentiation proceeds to osteocytic differentiation, just as in the skeleton, as shown by vascular expression of osteocyte specific genes such as SOST (gene for sclerostin) and E11 (gene regulating osteocytogenesis) (Staines et al., 2016; Bisson et al., 2019). SOST expression in VSMC subtypes was confirmed by single cell transcriptome analysis in aortas of high fat fed mice (Kan et al., 2021). The source of the osteoblastic differentiating cells may be mesenchymal progenitors from the adventia moving into the media and expressing both smooth muscle and osteoblastic transcriptional programs, Kramann et al. (2016) although other lineage tracing studies support vascular smooth muscle cell transdifferentiation (Speer et al., 2009; Naik et al., 2012; Durham et al., 2018).

In osteoblastic differentiation, Wnt/β-catenin signaling is critical for differentiation from mesenchymal stem cells, Gaur et al. (2005) and it induces RUNX2, a master transcription factor of the osteoblast transcriptome (Franceschi and Xiao, 2003; Komori, 2010; Cai et al., 2016). RUNX2 regulates osteoblastic differentiation through stimulating expression of several bone-important proteins, such as osterix, osteocalcin, alkaline phosphatase and sclerostin. RUNX2 is critical for VSMC transdifferentiation, and in turn is epigenetically regulated, also critical in VSMC transdifferentiation (Xia et al., 2015; Liu et al., 2021; Li et al., 2022). Sclerostin expression by inhibiting Lrp5/6, the co-receptors with Frizzled for Wnts, antagonizes canonical Wnt signaling providing a feedback break on RUNX2 induction and RUNX2 mediated VSMC transdifferentiation (Li et al., 2005).

In early CKD, the increase in circulating Wnt inhibitors directly affects the skeleton through Wnt inhibition, de Oliveira et al. (2013) but since Wnt signaling induces RUNX2, the Wnt inhibitors cannot be the basis for the vascular RUNX2 expression. RUNX2 is Wnt stimulated, and Wnts and RUNX2 are at the start of vascular osteoblastic/osteocytic transdifferentiation. So how does CKD activate vascular Wnt signaling? There are other signals from kidney disease, besides the Wnt inhibitors, activating vascular Wnts or other pathways leading to RUNX2 expression.

The transcriptome stimulated by vascular RUNX2 includes alkaline phosphatase, an important contributor to the CKD-induced decrease in inhibitors of calcification since it catabolizes pyrophosphate. Other inhibitors of calcification diminished in the circulation during CKD include fetuin-A (Shroff et al., 2008). Fetuin-A, synthesized in the liver, is glycoprotein that binds calcium in calciprotein complexes and prevents calcium salt crystallization (Heiss et al., 2010). Other inhibitors of calcification during CKD, such as osteopontin and MGP (matrix gla protein), are more complex than mere association with their circulating levels. MGP is discussed below in the skeletal section on Gla proteins. Osteopontin is a very complex protein in regards to vascular calcification. It is highly phosphorylated (thus Ca binding), exists as multiple splice variants with different functionality, is a cytokine stimulating the immune system, has an RGD integrin and CD44 binding domain, and is a SIBLING protein. SIBLING proteins (small integrin-binding ligand, N-linked glycoprotein) are components of the extracellular matrix of bone. There they play key roles in the mineralization of the tissue. Osteopontin is upregulated in calcified vessels as part of osteoblastic differentiation (Shen and Christakos, 2005), but serum levels do not correlate with calcific burden (Giachelli et al., 2005; Barreto et al., 2011). Osteopontin null mice have increased vascular calcification (Speer et al., 2002). *In vitro* and *in vivo* studies suggest that osteopontin not only inhibits mineral deposition but also actively promotes its dissolution by physically blocking hydroxyapatite crystal growth and inducing expression of carbonic anhydrase II in monocytic cells and promoting acidification of the extracellular milieu (Steitz et al., 2002). Despite its protective role in vascular calcification, circulating osteopontin has been implicated in the lung disease stimulated by kidney injury (Khamissi et al., 2022).

**FIGURE 4:**
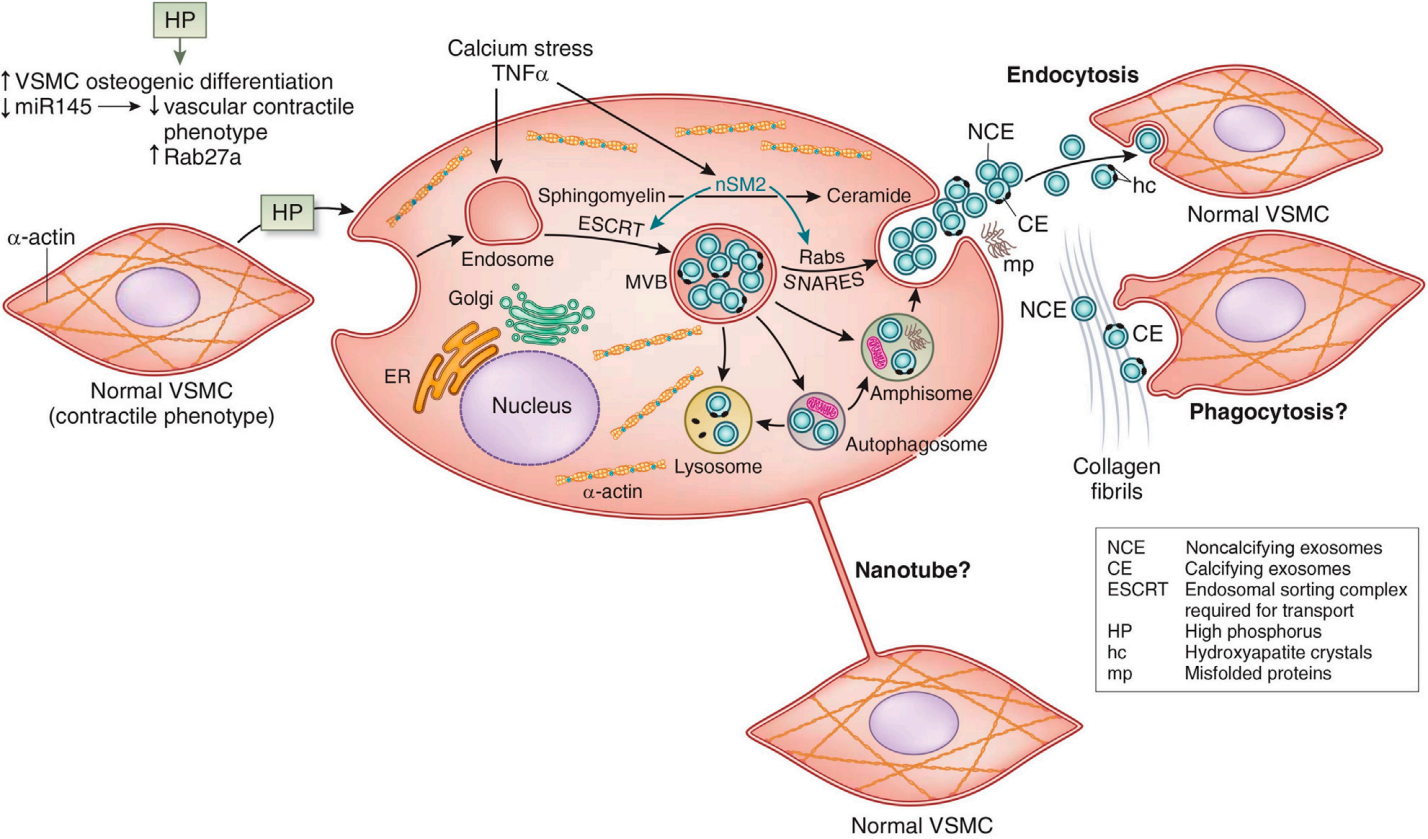
Vascular smooth muscle cell (VSMC) exosome biogenesis and release. See text for interpretation. miR145, micro RNA 145; ER, endoplasmic reticulum; TNF-α, tumor necrosis factor alpha; MVB, multivesicular bodies; nSM2, neutral sphingomyelinase 2; SNARES (Soluble NSF Attachment protein Receptor Superfamily). Adapted from Dusso et al. (2018) with permission.

In VSMC transdifferentiation, there is an increase in the release of matrix vesicles, which resemble those released by osteoblasts (Kapustin et al., 2015). Matrix vesicles are a form of extracellular vesicle implicated in both apoptosis and the calcification environment (Figure 4). Schurgers et al. (2018) They colocalize with collagen and elastin fibrils, and they have phospholipid membranes containing molecular cargo (protein, RNA, or lipid). Some of the matrix vesicles are exosomes formed by the endosomal pathway through multivesicular bodies (MVB) (Figure 4). In the pathway of CKD stimulation of osteoblastic exosome production, tumor necrosis factor α (TNFα) stimulates neutral sphingomyelinase 2 (nSM2) (Figure 4). Many cells and cell types release matrix vesicles in response to cell activation or apoptosis. These vesicles act serve as nucleation sites in calcification. They have the capacity for extracellular matrix production. Evidence suggests that these vesicles may permit cross-talk among VSMCs and between endothelial cells and VSMC (Lin et al., 2016; Chen et al., 2018a). This may support bidirectional positive feedback between intimal and medial calcification (Bardeesi et al., 2017). Also, nanotubes bridging cells are another potential mechanism of cell to cell communication in calcification (Figure 4). Vesicles that contain genotypic and/or phenotypic information such as microRNA, may either induce or inhibit nearby pro-calcific phenotypic change depending on the sequences released by VSMC (Goettsch et al., 2013; Dusso et al., 2018). Changes in relative collagen composition and the degradation of elastin produced by the vesicles may promote hydroxyapatite formation and provide scaffolding for further mineralization (Pai and Giachelli, 2010; Hodroge et al., 2017).

Besides matrix vesicles, apoptotic cell bodies can act similarly (Proudfoot et al., 2000). Furthermore, under the stress of CKD, autophagy is impaired, promoting DNA damage and leading to increased apoptosis (Tang et al., 2020). CKD is a state of early senescence, which impairs autophagy, promotes apoptosis and vascular calcification (Dai et al., 2019). In CKD, increased activin may affect negatively vascular autophagy through inhibition of MTORC2 (Chang et al., 2020).

Clinically, abnormalities of the vasculature found in early CKD, produce vascular stiffness and contribute to left ventricular hypertrophy (Mitsnefes et al., 2005; Ix et al., 2009; Seifert et al., 2013). Vascular calcification further intensifies stiffness and development of left ventricular hypertrophy, all processes that contribute to cardiovascular risk and excess cardiac mortality. The surprising fact is that despite many clinical trials targeting vascular calcification, there has been modest at best attenuation of the process and no demonstration of improved cardiovascular outcomes (Figure 2). Raggi et al. (2020), Xu et al. (2022) Thus, there is dire need for refocus on the therapeutic target, and new therapeutic approaches.

### Skeletal

Abnormalities of bone in the CKD-MBD begin with the renal response to injury due to activation of Wnt pathways in the kidney. Surendran et al. (2004), Zhou et al. (2016) Canonical Wnt signaling transcriptionally stimulates production of DKK1, Aaron (2001), Niida et al. (2004), Fang et al. (2014a) and DKK1 and SOST are both downstream targets of TGFβ family signaling activated by renal injury (Figure 3). Kamiya et al. (2010) Since the Wnt inhibitors are circulating factors, kidney injury directly inhibits bone remodeling which is homeostatically Wnt dependent. Bafico et al. (2001), Gong et al. (2001), Little et al. (2002) Inhibition of Beta catenin signaling in the skeleton, increased SOST expression, and increased expression of osteocytic FGF23 are seen after a relatively mild reduction in the glomerular filtration rate (GFR), as seen in stage 2 CKD. Pereira et al. (2009), Graciolli et al. (2010), Sabbagh et al. (2012) These abnormalities predominately affect cortical bone where osteocytes reside in early CKD, and progress to decreased cortical bone volume and porosity. Malluche et al. (2013), Nickolas et al. (2013), Fang et al. (2014b) Abnormalities of remodeling, mineralization and the material properties of bone develop in CKD, leading to major decreases in structural strength, fractures and deformity associated with long term disease. Moe and Nickolas (2016), Damasiewicz and Nickolas (2018), El-Husseini et al. (2022) Recent studies demonstrate the progressive development of a remodeling imbalance during CKD due to stimulation of osteoclast number and function, but a relative decrease in osteoblast function in view of increased osteoblast number that fails to increase bone formation as expected. Sugatani et al. (2017), Williams et al. (2018) This is fueled by acidosis and hyperparathyroidism in collaboration with activin/activin receptor type II signaling. Williams et al. (2018), Levy et al. (2023) The progression of the skeletal remodeling disorder in CKD to the familiar high turnover state related to hyperparathyroidism begins from a low turnover state produced by CKD induced Wnt inhibition in early CKD. Graciolli et al. (2010), Sabbagh et al. (2012), Fang et al. (2014a) The effect of PTH to stimulate remodeling in CKD is in part due to suppression of osteocyte SOST expression (Figure 3). SOST is regulated by a distal enhancer, ECR5, a 255-base pair evolutionarily conserved sequence within the von Buchanan deletion region. ECR5 contains a response element for the MEF2 family of transcription factors (MEF2A–D) (Leupin et al., 2007). In mice, deletion of Mef2c in the osteoblast/osteocyte lineage cells results in a high bone mass phenotype, suggesting that MEF2C is a major regulator of ECR5 dependent SOST expression (Collette et al., 2012). The class IIa histone deacetylase, HDAC5, is a negative regulator of MEF2C driven SOST expression in osteocytes (Wein et al., 2015). PTH stimulation of HDAC5 is a mechanism of its inhibition of SOST expression (Leupin et al., 2007).

Included in the abnormalities of skeletal remodeling and mineralization in CKD are disorders in the non-collagenous bone matrix proteins, matrix Gla protein (MGP) and osteocalcin (OC). MGP and OC belong to the mineral-binding Gla-protein family. These proteins contain a variable number (from 3 in osteocalcin to 5 in MGP) of glutamic acid (Gla) residues. Gla residues undergo γ-carboxylation making the proteins active in calcium binding. γ-Carboxylation is vitamin K–dependent. Undercarboxylated Gla proteins are less active.

OC is an abundant non-collagenous protein of bone. Its association with bone remodeling rates has made it a biomarker of renal osteodystrophy. OC is involved with the mineralization process through modulation of the shape and size of hydroxyapatite crystals. The mineralization function of OC requires activation through vitamin K–dependent carboxylation of Gla residues. Additionally, OC also modulates osteoblast and osteoclast activity, interacting with the G-protein–coupled receptor 6A (GPRC6A). Hormonal activity of OC was discovered by the finding that OC null mice develop a type 2 diabetes-like phenotype. The concept is that under-carboxylated OC (ucOC) acts as a hormone to increase pancreatic β-cell growth, insulin secretion, and insulin sensitivity. However, this concept is controversial and confirmatory research has not uniformly supported it. Karsenty (2020); Manolagas (2020) Circulating ucOC originates from bone, where osteoclastic bone resorption produces under carboxylation and renders the molecule able to reach the circulation. Plasma levels of OC have been linked with glucose homeostasis in type 2 diabetes and with metabolic syndrome (Saleem et al., 2010). These data suggest that bone is not only essential in mineral metabolism, but also regulates glucose and energy metabolism. This may be true independent of whether ucOC is a hormonal factor.

Besides bone, MGP is synthesized in several tissues, including VSMCs. Mice lacking MGP have intense medial calcification, demonstrating the role of MGP in VC (Luo et al., 1997), The mechanism of MGP and OC action include inhibition of calcium phosphate recipitation, regulation of osteoblastic differentiation, VSMC transdifferentiation, and regulation of osteoblast and VSMC-derived matrix vesicles (MVs), which, in turn, affect calcification. MGP binds to bone morphogenetic protein (BMP)-2, thus inhibiting the osteo-inductive properties of this cytokine (Wallin et al., 2000). MGP prevents VSMC apoptosis, and apoptotic bodies induce calcification. Studies in CKD-5D patients show increased levels of under carboxylated MGP (ucMGP, ie, non-functional) (Westenfeld et al., 2012). ucMGP correlated significantly with mineralization and cardiovascular disease in CKD (Cranenburg et al., 2008).

### Cardiac

Cardiac hypertrophy is highly prevalent in CKD. We found in a cohort of patients with CKD stage 3 from a single center clinic and a mean eGFR of 50 mL/min/1.73m2, an 80% incidence of left ventricular hypertrophy (LVH) by CT scanning (Seifert et al., 2014). This is in concert with larger population studies (Foley et al., 1998; Di Lullo et al., 2015). Cardiac hypertrophy is an early stage of cardiovascular disease leading to high rates of heart failure, sudden death, and even ischemic myocardial infarction (Foley et al., 1998). The causes of cardiac hypertrophy in CKD are multifactorial including vascular stiffness, FGF23, Gutierrez et al. (2009) valvular calcification and decreased αklotho - all components of the CKD-MBD (Figure 1). Increased vascular stiffness is a result of vascular calcification. The high levels of FGF23 causing cardiomyocyte hypertrophy are also late in CKD compared to the early elevations in FGF23 referred to above and elsewhere in this review (Chmielewski et al., 2009). Cardiac valvular calcification is not distinct from the vascular calcification pathogenesis discussed above in terms of the role of osteoblastic differentiation of cells in the valvular interstitium (Marwick et al., 2019). However, the CaLIPSO study found a major difference in SNF472 effect between the aortic valve and thoracic aorta, perhaps challenging this concept (Raggi et al., 2020). The role of αklotho in cardiac hypertrophy is discussed below in the FGF23 and αklotho sections.

## Endocrine components of the chronic kidney disease-mineral bone disorder: Fibroblast growth factor 23, klotho, calcitriol, parathyroid hormone (PTH)

### FGF23 (fibroblast growth factor 23)

FGF23 was discovered as the cause of autosomal dominant hypophosphatemic rickets in 2000 (ADHR Consortium, 2000). Shortly, thereafter it was shown to be dysregulated in CKD (Fliser et al., 2007). Its circulating levels strongly associate with mortality and CV outcome in CKD (Gutierrez et al., 2008; Isakova et al., 2018). Furthermore, the elevations in FGF23 begin prior to abnormalities in Ca, Pi, calcitriol and PTH in early CKD (Isakova et al., 2011). Most FGF-23 is produced by skeletal osteoblasts and osteocytes. The mediators of its release remain incompletely understood (Martin et al., 2012). Associations of FGF23 levels with PTH (Kawata et al., 2007), iron deficiency (Wolf and White, 2014), calcium (David et al., 2013), and vitamin D, Liu et al. (2006) have been consistently observed. The main functions of FGF-23 are to regulate phosphate and calcium homeostasis by stimulating urinary phosphate excretion and decreasing calcitriol levels (Wolf, 2012). The activity of FGF-23 to regulate phosphate homeostasis in the kidney requires the presence of αklotho, a co-receptor that facilitates binding of FGF-23 to the FGF-receptors (FGFR-1-3) (Cancilla et al., 2001; Chen et al., 2018b). FGF-23 excess is associated with poor outcomes in CKD, but its role in vascular calcification is less clear. Multiple studies have produced conflicting results. Human and animal VSMCs exposed to FGF-23 in the presence or absence of αklotho, and in the presence of normal or high phosphate, reveal increased, decreased, or no effect at all on mineralization of the matrix (Moe and Nickolas, 2016; Sugatani et al., 2017; Damasiewicz and Nickolas, 2018; El-Husseini et al., 2022; Levy et al., 2023). These conflicting results suggest a complex role of FGF-23 in vascular calcification, or the possibility that FGF-23 is not a causal factor.

FGF23 may be a causal factor in the LVH associated with CKD. Faul et al., demonstrate in studies *in vitro* and *in vivo* that FGF23 stimulates cardiomyocyte hypertrophy through the FGFR4 independent of klotho (Faul et al., 2011; Grabner et al., 2015). However, following the studies of Chen et al. (2018b) showing that soluble klotho was a co-receptor for FGF23 this concept has been modified, and it is discussed below in the αklotho section. It appears that the function of FGF23/FGFR4 is dependent on the co-receptor. When co-receptor function is served by heparin sulfate, myocyte hypertrophy is stimulated through activation of NFATC1, but if klotho serves as the co-receptor, hypertrophy is not stimulated (Leifheit-Nestler et al., 2021).

### αklotho (hereafter klotho)

In 1997, Kuro-o et al., reported a klotho hypomorph mouse displaying a phenotype of shortened lifespan, multiple organ degeneration, vascular calcification (VC), cardiovascular disease (CVD), abnormal phosphate and mineral regulation and frailty, similar to that of premature aging in humans (Kuro-o et al., 1997). Klotho is produced as a transmembrane protein of renal epithelial cells, with a large extracellular domain and only a few intracellular amino acids. Klotho serves as the cofactor for fibroblast growth factor 23 (FGF-23) to bind to its cognate receptor and regulate phosphorus and vitamin D metabolism (Kurosu et al., 2006; Urakawa et al., 2006). Cleavage of the extracellular domain produces the soluble form of klotho, which has been shown to be an endocrine factor. Klotho has antiaging properties, which are mediated by multiple systemic effects including regulation of insulin signaling, Wnt signaling, and prevention of vascular calcium deposits, oxidative stress, and fibrosis (Kuro-o et al., 1997; Kurosu et al., 2005; Doi et al., 2011; Hu et al., 2011). The kidney has the highest levels of klotho expression and is the major source of soluble klotho (Lindberg et al., 2014; Hu et al., 2016) by proteolytic cleavage of the transmembrane form as well as alternative gene transcription (Imura et al., 2004). CKD has been reported as a condition of klotho deficiency, with animal models of CKD demonstrating decreased klotho gene expression, lower klotho levels in kidney tissue, and lower circulating soluble klotho (Asai et al., 2012; Sakan et al., 2014). The decrease in renal Klotho in CKD may be stimulated Wnt signaling activation. This suggests the design of new strategies directed to increase Klotho levels could be considered as a strategy to reduce morbidity and mortality associated with kidney and heart diseases. Recent studies of a peptide from the KL1 domain of klotho show that it inhibits formation of TGFβ signal complex and decreases renal fibrosis confirming previous studies of sKL actions (Yuan et al., 2022).

Regarding vascular calcification, unlike FGF-23, evidence *in vitro* and *in vivo* support a protective role of klotho. (Toussaint et al., 2020). Klotho directly suppressed Pit-1 and Pit-2 activity and subsequently prevented phosphate-induced osteogenic transdifferentiation when added to a rodent VSMC line. In addition, models of klotho deficiency demonstrate increased expression of Pit-1 and Pit-2 receptors. This suggests that the klotho deficiency observed in CKD might result in upregulation of these receptors, promoting phosphate uptake and driving VSMC transdifferentiation.

Soluble Klotho protects against cardiomyopathy in CKD independent of FGF23 and phosphate (Xie et al., 2015). The mechanism may be through downregulation of TRPC6 channels in cardiomyocytes (Xie et al., 2012). However, recent studies show that the FGF23/FGFR4 stimulation of cardiomyocyte hypertrophy is dependent on co-receptors (Yanucil et al., 2022). FGF23/FGFR4/heparin sulfate complexes stimulate hypertrophy, while FGF23/FGFR4/klotho complexes inhibit it. Yanucil et al. (2022) All These studies have led to the development of recombinant klotho and klotho derived peptides as therapeutic approaches to progression of CKD and associated cardiomyopathy (Zhou et al., 2015; Hu et al., 2017; Yuan et al., 2022). The Alpha Young LLC is a biotech start up developing a klotho mimetic as a CKD therapeutic agent. However, as Isakova et al. point out, pharmacological properties of the agents, including bioactivity and stability, need to be improved, and the mechanism of action still needs further elucidation (Isakova et al., 2022).

### Calcitriol, vitamin D

Because of the elevations in FGF23 in early CKD, and its function to stimulate hydroxylation of 25-hydroxy vitamin D to 24,25- dihydroxy vitamin D in the catabolic pathway, vitamin D deficiency is a uniform complication of CKD. In addition, inhibition of proximal tubular 1alpha hydroxylase by FGF23 produces calcitriol (1,25 dihydroxy cholecalciferol) deficiency late in the course of CKD and promotes hyperparathyroidism. Calcitriol deficiency and secondary hyperparathyroidism were central components to the CKD-MBD, even before the syndrome was coined, and are critical to the development of renal osteodystrophy.

The effects of calcitriol are protean. It is a steroid hormone regulating cellular function through the nuclear vitamin D receptor (VDR), and VDR response elements (VDRE) in the promotors of many genes. This includes the PTH gene in which the VDRE serves to decrease transcription. Its actions on vascular calcification are “U-shaped”, with both low and high levels promoting medial vascular calcification. Zittermann et al. (2007) Calcitriol regulation of osteocyte sclerostin production is a factor in its actions on vascular calcification (Figure 3). Nguyen-Yamamoto et al. (2019) Osteocyte specific calcitriol deficiency blocked CKD stimulation of sclerostin levels, increased BMP2 in the skeleton, and increased CKD stimulated VC. Recent studies suggest that vitamin D deficiency in CKD induces reductions in aortic microRNA-145 stimulating osteogenic differentiation along with hyperphosphatemia (Figure 4). Carrillo-López et al. (2022).

In tubulopathies producing the Fanconi syndrome, calcitriol deficiency is associated with childhood osteomalacia.

Calcitriol is thought to regulate cardiac hypertrophy, see Covic et al. for review.Covic et al. (2010) Observational studies have indicated that vitamin D treatment was associated with a significant reduction of cardiovascular death among dialysis patients, and a reduction in LVH (Shoji et al., 2004). However, the PRIMO clinical trial looked to analyze this effect in CKD, and benefit of an active Vit D analogue (paricalcitol) on LVH was not detected (Figure 2) (Thadhani et al., 2012). A modern non-linear Mendelian randomization study continues to show that vitamin D deficiency increases mortality (Sutherland et al., 2022).

### PTH

PTH is a central component of the pathogenesis of renal osteodystrophy and it is the major factor driving the high turnover remodeling state seen in late CKD and CKD treated with hemodialysis. The mechanism of transition from the early CKD low turnover state produced by Wnt inhibitors including sclerostin, to the high turnover state stimulated by PTH was discussed above in the skeletal section. PTH has also been implicated in vascular calcification, although the effects are complex. Several lines of evidence support a direct role of PTH. Elevated PTH is associated with higher rates of vascular atherosclerotic disease (Hagström et al., 2014). Animal models with synthetic PTH infusion develop extensive calcification independent of hypercalcemia (Neves et al., 2007). In subtotally nephrectomized rats, suppression of PTH by treatment with a calcimimetic or parathyroidectomy slows aortic calcification independent of serum calcium and phosphate concentrations (Kawata et al., 2008; Jung et al., 2012). Clinical trials using cinacalcet have not successfully affected vascular calcification or cardiovascular outcomes despite lowering PTH levels (Figure 2) (Raggi et al., 2011; Investigators et al., 2012).

Treatment of hyperparathyroidism in CKD, with use of calcium-containing phosphate binders, can result in low bone turnover and reduced mineralization. This may potentiate vascular calcification (Raggi et al., 2005). States of low bone turnover and reduced mineralization are likely to attenuate the skeletal capacity for effective calcium and phosphate buffering and homeostasis. A reduction in skeletal buffering capacity exposes the vasculature to greater fluctuations in extracellular calcium and phosphate, thereby increasing the propensity for vascular calcification (Persy and D'Haese, 2009).

## Mineral components of the chronic kidney disease-mineral bone disorder: phosphate (Pi), calcium (Ca)

The proximal tubule of the kidney regulates phosphate excretion. However, the balance of bone formation and resorption is also a contributor to serum levels (Mathew et al., 2008). Hormonal regulation of phosphate at the renal and gastrointestinal levels by parathyroid hormone (PTH), fibroblast growth factor-23 (FGF-23), klotho and 1,25-dihydroxyvitamin D (calcitriol) maintains phosphate levels in serum from 2.5 to 4.5 mg/dl. As impaired kidney function progresses to advanced stages, with reduction in functional nephron mass, phosphate excretion is impaired, PTH is stimulated, bone remodeling produces excess resorption, and together with persistent dietary intake, serum phosphate concentrations rise.

Multiple studies demonstrate the association between hyperphosphatemia and vascular calcification (Raggi et al., 2002; Adeney et al., 2009), especially in patients on hemodialysis (Block et al., 2004; Chertow et al., 2004). Since phosphate is a component of hydroxyapatite crystals, it is conceivable that increased serum levels alone in the context of CKD could contribute directly to precipitation of hydroxyapatite in tissue (Shanahan et al., 2011). Despite this possibility, not all patients with sustained hyperphosphatemia develop vascular calcification.

**FIGURE 5:**
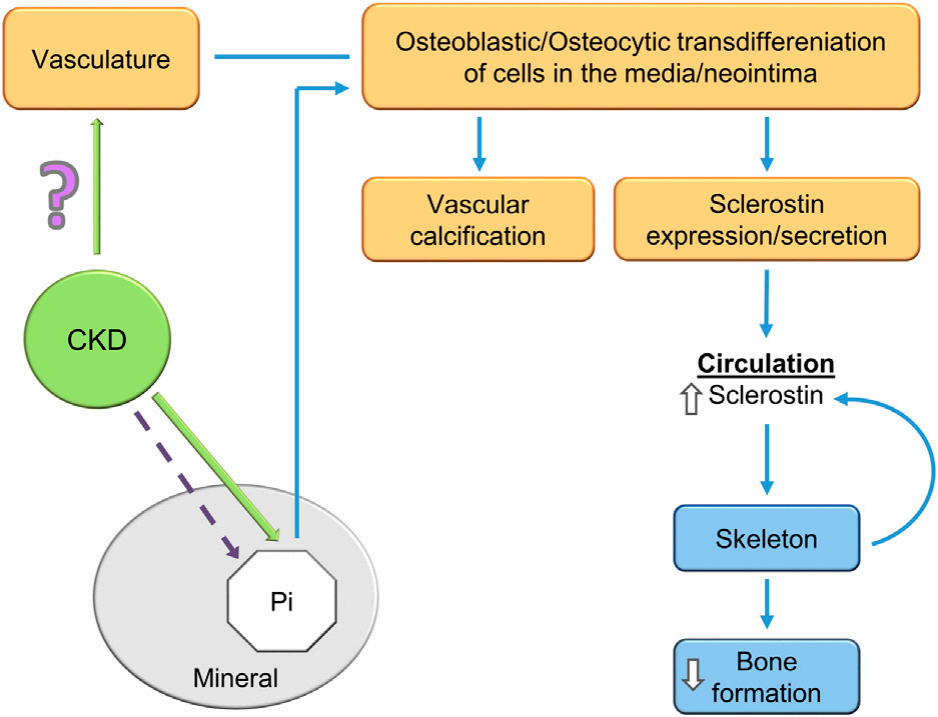
A potential explanation of the CKD-MBD bone-vascular paradox. CKD induces osteoblastic/osteocytic transdifferentiation of vascular smooth muscle cells. The osteoblastic/osteocytic cells stimulate vascular calcification and express high levels of sclerostin as shown by Mace et al. (2021); Mace et al. (2022) and others. Sclerostin secretion to the circulation decreases skeletal remodeling and inhibits skeletal calcification (an explanation of the paradox). Skeletal osteocytes also contribute to the elevated circulating sclerostin levels in CKD. How CKD stimulates VSMC transdifferentiation is unknown, although hyperphosphatemia is a contributor in the late stages of the syndrome.

Furthermore, hyperphosphatemia is an active stimulator of signal transduction leading to vascular calcification. Seminal observations *in vitro* demonstrated a process wherein exposure to elevated phosphate stimulated a dose- and time-dependent phenotypic change in vascular smooth muscle cells (VSMCs) (Jono et al., 2000; Steitz et al., 2001). Upregulation of phosphate transporters (Pit-1 and Pit-2) occurs in the context of hyperphosphatemia. Signal transduction from Pit-1 and Pit-2 mediate transdifferentiation of VSMCs to an osteochodrogenic cell phenotype (Figure 5) (Villa-Bellosta et al., 2007; Pai and Giachelli, 2010). The VSMC transdifferentiation is inhibited by competitive antagonism of Pit-1. Furthermore, phosphate-induced autophagy counteracts vascular calcification by reducing matrix vesicle release (Dai et al., 2013).

Phosphate overload downregulates renal Klotho expression through inhibition of peroxisome proliferator-activated receptor gamma (PPARγ) and the activation of the Wnt/β-catenin signaling pathway, thus contributing to the development of vascular calcification and the alteration of the regulation of mineral metabolism (Hu et al., 2013; Liu et al., 2018; Maique et al., 2020; Muñoz-Castañeda et al., 2020). Failure of clinical trials directed at hyperphosphatemia to affect vascular calcification may be related to the minimal to modest effect of interventions on hyperphosphatemia (Figure 2) (Toussaint et al., 2020; Ogata et al., 2021; Xu et al., 2022). However, an open label trial comparing sevelamer and calcium carbonate has detected an effect on all cause mortality, while others have failed (Di Iorio et al., 2012). Recent progress has lead to a FDA advisory committee recommendation of an NHE3 inhibitor (tenapanor) producing decreased intestinal sodium absorption and secondarily phosphate absorption. (Ardelyx press release 17 Nov 2022) The effect on serum phosphate is similar to that of phosphate binders, and tenapanor can be combined with phosphate binders for increased effect (Pergola et al., 2021). The effects of this approach on clinical outcomes will be forthcoming in the next years.

## The bone—vascular paradox and possible identification of the chronic kidney disease-mineral bone disorder therapeutic target along with a biomarker

The bone–vascular paradox (also referred to as the calcification paradox and the bone vascular axis) in the CKD-MBD consists of the observation that as vascular calcification (heterotopic mineralization) is stimulated, bone formation (orthotopic mineralization) is inhibited (Persy and D'Haese, 2009; Evenepoel et al., 2019). In this scenario, the worse the vascular calcification the greater the inhibition of skeletal remodeling (El-Husseini et al., 2022). In agreement with this concept, patients with the worst vascular calcification are often osteoporotic. Recent studies have provided insight and a possible basis of the paradox (Figure 5). Mace et al. used the novel model of aortic transplantation (Atx) in isogenic rats (Mace et al., 2021). Using aortas from rats with CKD and severe vascular calcification transplanted into normal rats (uremic Atx), they demonstrated decreased trabecular bone mineral density (BMD) 14 weeks following aortic transplantation (Mace et al., 2021). Bone histomorphometry analysis showed significant lower osteoid area in uremic ATx compared with normal ATx along with a trend toward fewer osteoblasts as well as more osteoclasts in the erosion lacunae. Furthermore, skeletal RUNX2, osteopontin, ANKH (progressive ankyloses protein), alkaline phosphatase, and type 1 collagen alpha 2 genes and proteins were induced in rats transplanted with calcified aortas from uremic rats. This is a key observation demonstrating that the calcified transplanted vasculature from rats with CKD regulated skeletal gene expression, transcription and bone resorption leading to decreased BMD. Transplantation of aortas from normal rats into normal rats produced no effects. Tissue culture of calcified aortic rings from rats with CKD revealed secretion of large amounts of sclerostin into the media, while the aortic ring cultures derived from normal rats did not secrete sclerostin. Studies *in vitro* showed that the calcified uremic aortic rings inhibited matrix mineralization by osteoblast cell lines compared to aortic rings from normal rats (Mace et al., 2022). The uremic calcified aortic rings stimulated the same gene expression and protein levels that were demonstrated in the experiments *in vivo*, and inhibited canonical Wnt/Beta catenin signaling and activated activin receptor signaling.

The high levels of sclerostin secretion from the calcified uremic aortas are in agreement with studies from other investigators, showing that sclerostin is expressed in association with vascular calcification vasculature (Zhu et al., 2011; Rukov et al., 2016; Bisson et al., 2019). This suggests that a significant portion of the elevated sclerostin levels in patients with CKD may derive from vascular and non-skeletal sources, (Zhou et al., 2017; Li et al., 2019). Although, not all human investigations have found high levels of vascular sclerostin (Qureshi et al., 2015) However, the strong direct association between circulating sclerostin levels and vascular calcification are much more a consensus (Qureshi et al., 2015; Zhou et al., 2017; Li et al., 2019). The studies raise the question of the role of vascular sclerostin (Claes et al., 2013) and FGF23 (Fang et al., 2014b) in the process of vascular calcification. The vascular expression of these mineralization inhibitors would be expected to be protective against vascular calcification, acting as a brake on Wnt stimulation of vascular RUNX2 and osteoblastic transdifferentiation (Figure 5).

**FIGURE 6:**
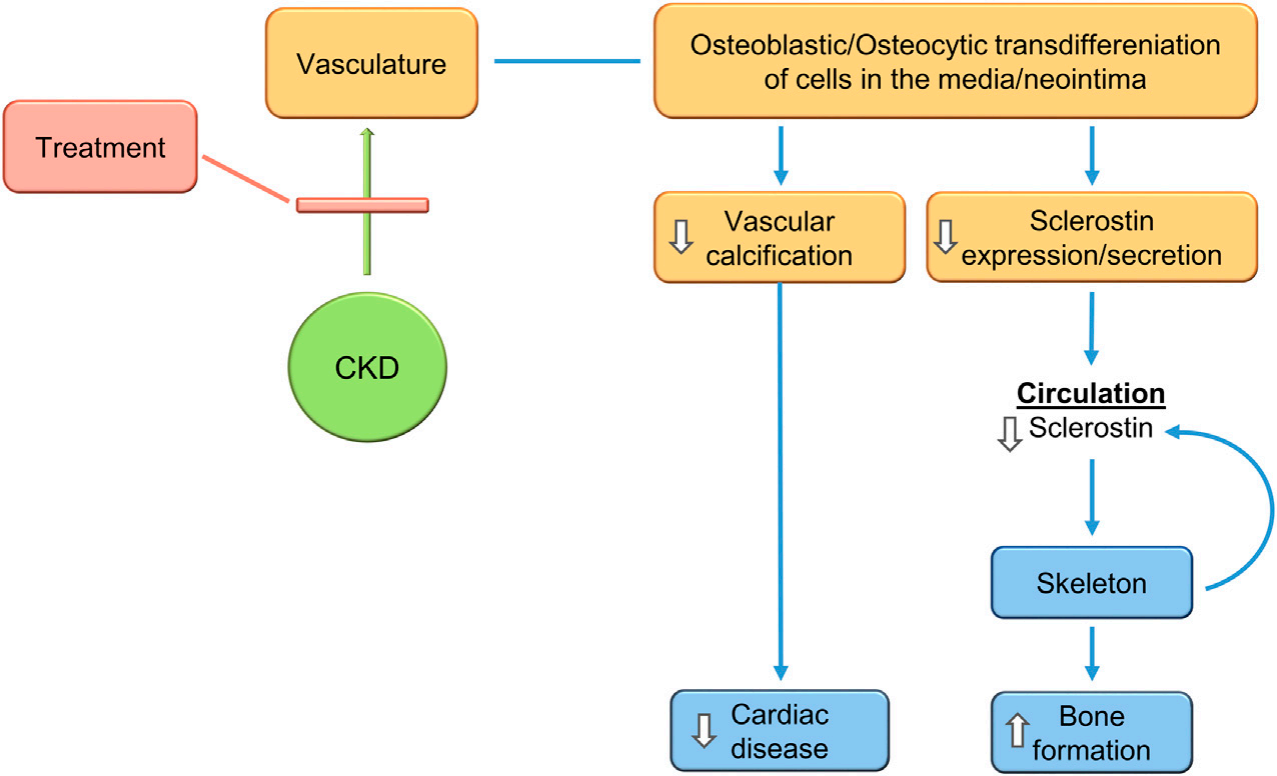
Treatment of the CKD-MBD inhibits VSMC transdifferentiation to osteocytic type cells, inhibiting vascular calcification, decreasing sclerostin secretion, and stimulating bone formation. This would produce expected efficacy in cardiovascular and skeletal outcomes. The changes in sclerostin levels during treatment make it a biomarker of progress.

Recent studies analyzing vascular calcification models in SOST deficient mice and sclerostin antibody treated mice confirm the expectations. De Mare et al., show that adenine-exposed Sost^−/−^ mice compared to adenine exposed wild type mice had much more CKD-induced cardiac vessel and renal artery calcification (De Maré et al., 2022). They also showed that the vascular calcification produced by the warfarin model of rat vascular calcification was intensified by treatment with a neutralizing antibody to sclerostin (De Maré et al., 2022). Thus, inhibition of sclerostin activity may not the objective of CKD-MBD treatment, rather reduction of sclerostin levels by inhibition of its production may be the goal (Figure 6).

The role of sclerostin in cardiovascular disease has been studied in other settings outside of CKD; see Golledge and Thanigaimani for an exhaustive review (Golledge and Thanigaimani, 2022). Developmentally, sclerostin is widely expressed in VSMC throughout the cardiovascular system, and postnatally, it is implicated in a number of vascular diseases. Despite some conflicting studies, the majority of animal models suggest that sclerostin deficiency increases aortic aneurysm, atherosclerosis, and cardiac rupture, and have found its upregulation at sites of arterial calcification.

Golledge and Thanigaimani state that “human studies report that genetic variants causing lower arterial sclerostin expression increase the prevalence of hypertension and diabetes and elevate the risk of cardiovascular events.” Other “human studies have associated high circulating sclerostin levels with the diagnosis of a variety of different cardiovascular diseases, surrogate markers of cardiovascular disease and high risk of cardiovascular events in some populations.” There are a number of possible explanations for the findings of human genetic studies, which suggest that low arterial sclerostin promotes cardiovascular disease, but human biomarker studies showing high serum sclerostin is associated with cardiovascular disease and events. One explanation is that the high blood sclerostin concentrations in participants with cardiovascular disease represent a response to the disease serving as a brake rather than a cause. The De Mare et al. studies discussed above support this concept (De Maré et al., 2022).

**FIGURE 7:**
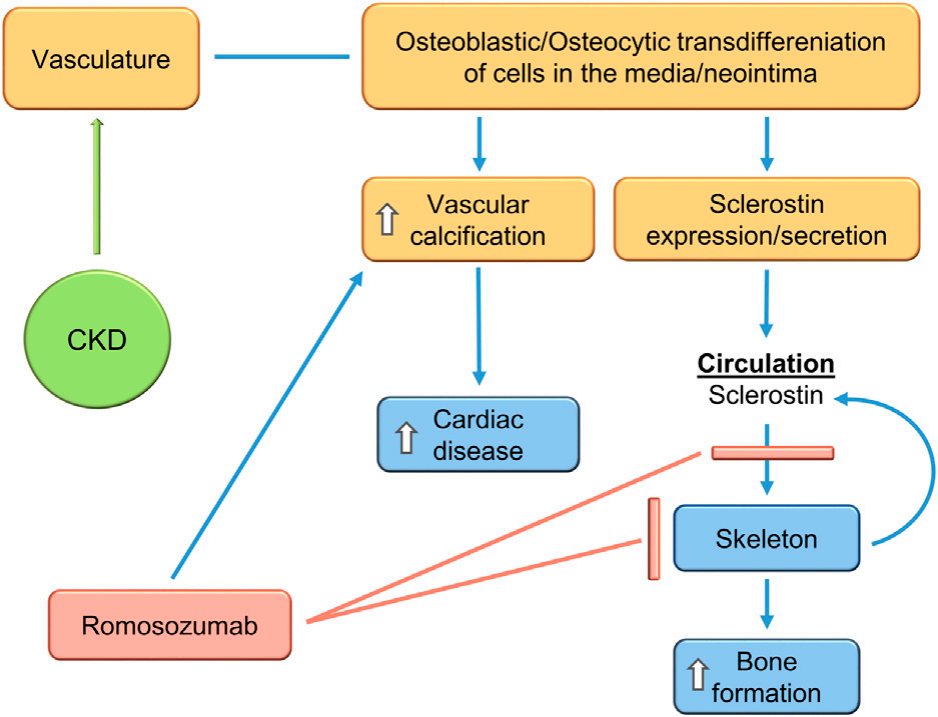
Treatment of the CKD-MBD using the antibody to sclerostin increases VSMC Wnt activity and osteoblastic transdifferentiation, stimulating vascular calcification, but blocking systemic and skeletal sclerostin effects, thus stimulating bone formation. This would produce expected efficacy in skeletal outcomes but worsen cardiovascular disease.

Romosozumab is an anti-sclerostin antibody approved for the treatment of osteoporosis, Cosman et al. (2016) but the label contains a black box warning about a potential increase in cardiovascular events. This has led to additional studies and controversy (Hólm et al., 2021). In a meta-analysis of randomized controlled trials of romosozumab, administration of the sclerostin blocking antibody may have increased the risk of major adverse cardiovascular events or cardiovascular death (Bovijn et al., 2020; Lv et al., 2020). Overall, currently available data as reviewed elsewhere, including the aforementioned metanalyses (Golledge and Thanigaimani, 2022) does not show conclusive evidence that sclerostin inhibition increases the risk of cardiovascular events. Limited data on the use of romosozumab in CKD patients on dialysis is emerging (Figure 7) (Sato et al., 2021; Saito et al., 2022). There is progression of vascular calcification, but no evidence of increased cardiovascular events, though the study sizes are small.

The structural properties of the 24 kDa sclerostin protein reveal a tertiary structure consisting of three loops, a cysteine-knot motif with three disulfide bonds and N- and C-terminal spacer arms (Wijenayaka et al., 2016; Omran et al., 2022). Sclerostin interacts with heparin sulfate mediating transportation to the cell surface and embedding in the extracellular matrix. Sclerostin interacts also with the co-receptors LRP4, 5, and 6 and thereby impedes the binding of mainly Wnt1 but also Wnt3a. Both Wnt1 and sclerostin interact with the E1 and E2 ectodomains of LRP6 (Boschert et al., 2013). This interaction inhibits directly the binding of Wnt1 and allosterically prevents the binding of Wnt3a to LRP6 and thereby prevents receptor-coupling with Frizzled on osteoblasts. Recent studies dissecting the functions of components of the sclerostin protein suggest that while the loop 3 peptide is inhibitory to the skeleton, it did not protect in an aortic aneurysm model (Yu et al., 2022). In the ApoE ^−/−^model of atherosclerosis, inhibiting the sclerostin loop 3 did not increase vascular calcification, but did increase bone remodeling (Yu et al., 2022). These studies, if confirmed, provide a new approach to targeting sclerostin with a therapeutic agent.

The idea that sclerostin might be a biomarker of the CKD-MBD was originally put forth by Cejka et al. (2011) and by others (Behets et al., 2017; Figurek et al., 2020; Omran et al., 2022). The recent studies clarify this potential role and clarify the path forward. Elevated sclerostin levels in CKD could indicate that VSMC transdifferentiation has occurred, and treatment showing a reduction in levels could indicate decreased vascular calcification and improved skeletal homeostasis. The multi-site production of sclerostin in CKD weakens the possibilities of a clear biomarker status, but future research needs to clarify the possibility. The clinical application of sclerostin levels has not yet occurred due in part to variability and lack of reproducibility between various sclerostin assays. The topic of sclerostin assay is beyond the scope of this review and has been analyzed in depth recently (Omran et al., 2022).

## Conclusion

The CKD-MBD is a complex multifactorial syndrome. Recent progress continues to clarify its pathophysiology, and suggests that a target of therapy would be to reduce the vascular injury associated with CKD. Reduction of vascular osteoblastic/osteocytic transdifferentiation would decrease, possibly prevent, vascular calcification and stimulate skeletal homeostasis (Figure 6). Reduction of sclerostin levels due to inhibition of osteocytic vascular transdifferentiation would be associated with reduced vascular calcification and improved bone remodeling in the CKD-MBD. Whereas, if significant portions of circulating sclerostin are of vascular origin in CKD, inhibition of sclerostin function by a monoclonal antibody would worsen vascular calcification while improving bone remodeling/formation (Figure 7). This suggests that the therapeutic target of the CKD-MBD may be osteoblastic/osteocytic vascular cell transdifferentiation with the primary outcome of clinical trials being cardiovascular events. Furthermore, sclerostin levels may be a useful biomarker, both diagnostically and as a means of following therapeutic efficacy. Successful studies in the treatment of the CKD-MBD would lead to studies proving or not, that CKD-MBD treatment independent of other mechanisms decreases the mortality associated with CKD.
